# Rapid Weight Reduction in Judo: Dietary Practices and Short-Term Health Effects

**DOI:** 10.3390/nu17243964

**Published:** 2025-12-18

**Authors:** Wiktoria Staśkiewicz-Bartecka, Paulina Ziomek, Daria Dobkowska-Szefer, Ewa Malchrowicz-Mośko, Paweł Tomaszewski

**Affiliations:** 1 Faculty of Public Health in Bytom, Medical University of Silesia in Katowice, ul. Jordana 19, 41-808 Zabrze, Poland; 2 Faculty of Physical Education, Józef Piłsudski University of Physical Education, ul. Marymoncka 34, 00-968 Warsaw, Poland

**Keywords:** rapid weight loss, judo athletes, nutritional behaviors, performance outcomes, combat sports nutrition

## Abstract

Background: Rapid weight loss (RWL) is a widespread practice among judo athletes seeking to compete in lower weight categories. Restrictive nutritional and behavioral strategies used to achieve short-term reductions in body mass may impair physiological and psychological functioning. This study aimed to assess dietary behaviors, weight-cutting methods, and short-term physical and psychological effects of RWL among competitive judo athletes. Methods: A cross-sectional study was conducted between August and December 2024 among 70 judo athletes (17 women, 53 men) competing at national and international levels. Data were collected using an author-designed questionnaire addressing anthropometric variables, training history, RWL strategies, dietary restrictions, hydration manipulation, and post-weigh-in eating behaviors. Physical and psychological symptoms were assessed using frequency-based self-report measures. Results: Most athletes (95.7%) reported engaging in RWL, typically beginning between ages 13 and 16 years (65.7%). Athletes reduced an average of 3.64 ± 1.74 kg (2–5% of body mass). Common strategies included decreasing meal frequency (74.29%), increasing training load (72.86%), restricting fluids (68.57%), and using saunas (62.86%). Reported physical symptoms included weakness (71.43%), headaches (51.43%), and dizziness (45.71%), while psychological symptoms included irritability (57.14%), reduced motivation (40%), and tension (38.57%). Post-weigh-in binge eating occurred in 65.71% of athletes and was significantly associated with higher RWL magnitude (*p* < 0.05). Discomfort during competition related to overeating (55.71%) or insufficient intake (41.43%) was also frequent. Conclusions: RWL is highly prevalent among judo athletes and often begins early in training history. The strategies used are associated with adverse physical and psychological symptoms and maladaptive compensatory behaviors. These findings highlight the need for evidence-based nutritional education and individualized weight-management approaches to support athlete health and performance.

## 1. Introduction

Weight reduction to qualify for a lower weight category is a widely used practice among combat sports athletes. In judo, as in other combat sports, weight categories are divided by gender [1]. For this reason, judokas often strive to compete against lighter and therefore potentially physically weaker opponents by reducing their own body mass in order to compete in a lower weight category and increase their chances of winning [2]. As part of competition preparation, combat sport athletes typically reduce their body mass by approximately 2–10% during the 2–3 days preceding the weigh-in procedure [3,4].

Among the methods used to achieve a lower body mass, both long-term reduction techniques and rapid weight loss are distinguished [4,5]. An optimal body mass management strategy should include both long-term and rapid components [6]. Rapid Weight Loss (RWL) is a commonly used practice before competitions. RWL is defined as a rapid reduction in body mass within a few days (up to 1 week) prior to the weigh-in procedure, achieved through various methods such as reduced food and fluid intake, restriction of specific food groups, and increased exercise intensity, often with additional clothing that enhances sweating [6,7,8]. A rapid weight-reduction strategy may be used as a method to decrease body mass in a short period of time; however, according to recommendations, total mass loss should not exceed 5%, and the time between weigh-in and the fight must be sufficient for refeeding and rehydration, i.e., more than 3 h [4].

Most of the body mass reduced within periods not exceeding 24 h results from manipulation of body fluids, which causes a rapid decrease in body mass [7,9]. To remove excess water from the body and thereby achieve weight loss, dehydration is a commonly used method among athletes. One approach to reducing total body water is lowering fluid intake below standard daily losses. In addition, athletes often use saunas, hot baths, or train in plastic suits, and some also use pharmacological diuretics [4].

Depleting glycogen stores constitutes another effective method of reducing body mass in combat sports. Methods intended to decrease glycogen stored in the body include implementing a low-carbohydrate diet with carbohydrate intake below 50 g per day during fight week while maintaining the previously established standard training plan, which leads to glycogen depletion and prevents its replenishment. Another glycogen-minimizing strategy used by athletes includes incorporating additional physical exercise to enhance depletion [4].

Another nutritional strategy is the reduction in gastrointestinal content volume. Implementation of a low-residue diet with minimal food mass for 24–96 h before the weigh-in procedure effectively reduces the mass of intestinal contents while simultaneously allowing athletes to maintain an adequate intake of energy and macronutrients [4].

It is important to emphasize that RWL techniques may have negative effects on both the physical and psychological condition of combat sport athletes [1,10,11]. Increased feelings of tension, anger, fatigue, and significant decreases in energy levels are among the symptoms experienced by athletes who rapidly lose body mass [10,12]. Therefore, it is crucial to highlight the importance of recovery after achieving the target weight among athletes who incorporate body mass reduction as part of their competition preparation. Minimizing the consequences of RWL and reversing the associated physiological disturbances are essential for athletes to benefit from body mass reduction in combat sports. Hydration, glycogen restoration, and minimizing gastrointestinal discomfort that may occur due to dietary modifications are the most important aspects of athlete recovery during the period between weigh-in and competition [9].

Although rapid weight reduction practices have been widely investigated in various combat sports, evidence focusing specifically on judo remains limited. Judo is characterized by a high frequency of competitions, short recovery periods between weigh-ins and bouts, and distinct technical and physiological demands, which may influence both dietary behaviors and weight-cutting strategies. Moreover, data examining gender-related differences in rapid weight reduction practices and their short-term physical and psychological consequences within a single judo-specific population are scarce, particularly in the Polish context. Therefore, the present study aims to address these gaps by providing a comprehensive analysis of dietary practices, frequency of rapid weight reduction techniques, and their immediate health-related outcomes among male and female judo athletes, thereby contributing novel, sport-specific insights to the existing literature.

This study allows for the identification of techniques most commonly used among judokas and for comparing them with the physical and psychological well-being of athletes both during their application and during the fight. This aspect is important due to the risk of negative health consequences resulting from restrictive weight reduction methods practiced by combat sport athletes. The study findings may serve as a tool to support the selection of RWL techniques in combat sports by increasing athletes’ and coaches’ awareness of their potential effects. This will allow the planning of nutritional strategies that minimize the risk of adverse outcomes of RWL that may influence fight performance.

The aim of this study was to analyze the nutritional behaviors of judo athletes used to achieve a body mass corresponding to a lower weight category, as well as the consequences of these practices, including the occurrence of compensatory behaviors after official weigh-in. It was hypothesized that the nutritional behaviors and rapid weight reduction practices used by judo athletes before competitions are associated with the occurrence and increased frequency of specific short-term physical symptoms (including reduced energy levels, weakness, dizziness, gastrointestinal discomfort, and dehydration-related complaints) as well as psychological effects (such as irritability, low mood, concentration difficulties, and appetite dysregulation), as assessed using a structured questionnaire. Furthermore, it was assumed that these effects differ according to sex.

## 2. Materials and Methods

### 2.1. Procedure for the Study

The data for the study were collected between August and December 2024. The study included judo athletes competing at national and international levels. A purposive sampling strategy was applied, which allowed for the selection of participants whose experience in the sport best aligned with the scope of the research. This approach was essential for obtaining reliable results concerning the specific characteristics of judo.

Nutritional practices aimed at rapid weight loss in the pre-competition period were assessed using an author-designed questionnaire. Participation in the study was voluntary, and completing the questionnaire was considered equivalent to providing informed consent, as indicated to participants at the beginning of the form. Data collection was conducted via the Google Forms platform due to its accessibility and ease of use. The Google Forms questionnaire was distributed directly to judo athletes, who represented the target population of the study. The World Medical Association’s Declaration of Helsinki guided the conduct of this study. The study was approved by the Bioethics Committee of the Silesian Medical University in Katowice (BNW/NWN/0052/KB/229/23, approved on 25 October 2023) in light of the Law of 5 December 1996 on the Profession of Physician and Dentist (Journal of Laws 2016, item 727).

### 2.2. Participants

A total of 70 individuals participated in the study. The study group comprised 17 women (24.3%) and 53 men (75.7%). The age of the participants ranged from 16 to 27 years. All respondents were residents of Poland. Each participant was informed about the complete anonymity of the study, the impossibility of linking responses to their identity, as well as the purpose and manner of data use.

The inclusion criteria were: (1) age of at least 16 years, (2) provision of informed consent to participate in the study, and (3) active training in judo with a minimum of one competition start per year.

Participants were excluded if they: (1) did not consent to participate in the study, (2) completed the questionnaire incorrectly or incompletely, or (3) had been training judo for less than one year.

It should be noted that judo training in Poland typically begins at an early age (often between 5 and 7 years), particularly among athletes who later compete at national and international levels; therefore, a long training history among relatively young participants is realistic and consistent with established athlete development pathways in this sport.

### 2.3. Survey Tools

The study was conducted using an author-designed questionnaire consisting of 24 items aimed at gathering information essential for achieving the study objectives. The first seven questions enabled the collection of demographic data used to characterize the participants, including sex, age, height, body mass, subjective assessment of ideal body weight, current competitive weight category, and years of judo training experience.

The study employed an original, author-designed questionnaire developed specifically for the assessment of rapid weight reduction practices, dietary behaviors, and associated short-term physical and psychological effects among judo athletes. The questionnaire was constructed based on a comprehensive review of the literature on weight-cutting strategies in combat sports and consultations with researchers experienced in sports nutrition and combat sports, ensuring content relevance and clarity. Prior to the main study, the questionnaire was pilot-tested on a small group of judo athletes to evaluate comprehensibility, wording, and feasibility of completion; minor modifications were introduced based on participant feedback. Given the exploratory nature of the study, a full psychometric validation was not conducted, which is acknowledged as a limitation.

The nutritional status of the participants was assessed using the body mass index (BMI), calculated according to the formula recommended by the World Health Organization (WHO). The results were interpreted in accordance with WHO classification criteria, as presented in Table 1.

The remaining 17 questions were directly aligned with the aims of the study. They addressed various aspects of pre-competition preparation, including the use of weight reduction techniques, the age at which athletes began practicing them, the number of kilograms reduced to reach the competition weight, and the number of days prior to competition when athletes initiate RWL techniques before the weigh-in procedure. Questions related to pre-competition nutrition referred to foods restricted or consumed in increased amounts during the reduction period, the types of fluids most frequently consumed, and the perceived frequency of physical and psychological effects of rapid weight loss. Items addressing nutrition between weigh-in and competition focused on commonly consumed foods and fluids, the occurrence of post-weigh-in binge eating, the presence of associated discomfort, its manifestations, as well as the frequency and type of symptoms linked to insufficient energy intake.

The questionnaire included open-ended questions, closed-ended single- and multiple-choice questions, as well as closed-ended items with an option to provide an additional response. All questions were formulated to ensure clarity and unambiguity for respondents. 

The practices under investigation were evaluated in terms of their frequency and subsequently compared between female and male athletes.

### 2.4. Statistical Analysis

Statistical analyses were performed using Statistica v.13.3 (StatSoft, Kraków, Poland) and the R package v. 4.0.0 (2020) under the GNU GPL (The R Foundation for Statistical Computing, Vienna, Austria). Measurable variables were analyzed using descriptive statistics. Their presentation included arithmetic mean, standard deviation, median, as well as minimum and maximum values. The frequency distribution of non-measurable variables was examined using multiway tables and reported as absolute numbers and percentages.

The normality of variable distributions was assessed using the Shapiro–Wilk test. Homogeneity of variances was evaluated prior to the application of parametric tests. Differences between male and female athletes were analyzed using the independent-samples Student’s *t*-test for variables meeting the assumptions of normality and equal variances. When the assumption of normality was violated, the non-parametric Mann–Whitney U test was applied. Associations between categorical variables were examined using the chi-square test of independence.

Associations between variables were assessed using non-parametric measures appropriate for the level of measurement. The Gamma correlation coefficient was applied for analyses involving ordinal variables, as it is suitable for assessing the strength and direction of monotonic relationships between ranked categories. Cramér’s V coefficient was used for associations between nominal variables, as it provides a standardized measure of association strength for categorical data derived from chi-square statistics.

A power-based rationale was provided assuming α = 0.05 and 80% power (two-sided). With the present unequal group sizes (men *n* = 53; women *n* = 17), the study is adequately powered primarily for large effects (minimum detectable effect for 80% power: d ≈ 0.79). For chi-square tests with df = 1, approximately N ≈ 88 would be required to detect a medium association (Cohen’s w = 0.30).

Results were considered statistically significant at *p* < 0.05.

## 3. Results

### 3.1. Sample Characteristics

Table 2 presents the aggregated anthropometric characteristics of the study participants, including age, height, body mass, and BMI, reported for the total sample as well as separately for men and women. No statistically significant differences were observed between sexes in terms of age, height, or BMI (independent-sample Student’s *t*-test, *p* > 0.05). A significant difference was identified only for body mass (independent-samples Student’s *t*-test, *p* < 0.001), which reflects natural sex-related variation in this parameter. The majority of participants exhibited normal body weight (75.72%, *n* = 53). Overweight status was identified in 18.57% (*n* = 13) of the respondents, obesity class I in 4.29% (*n* = 3), and underweight in 1.43% (*n* = 1). No statistically significant sex differences were found in the distribution of BMI categories (chi-square test of independence, *p* > 0.05).

The mean training experience among the athletes was 13.43 ± 3.39 years. The longest reported training history was 20 years, while the shortest was 2 years. No statistically significant differences in training experience were observed between men and women (Table 2).

### 3.2. Nutritional Strategies for Rapid Weight Reduction Used During Competition Preparation

Almost all participants (95.71%, *n* = 67) reported practicing weight reduction as part of their competition preparation, whereas only three athletes (4.29%) declared not engaging in weight-cutting before competitions. No statistically significant differences were found between men and women regarding the use of weight reduction strategies (chi-square test of independence, *p* = 0.71). On average, athletes reduced 3.64 ± 1.74 kg to compete in a lower weight category. In the analysis of weight-loss ranges, the most frequently observed reduction was 5–7% (31.43%), whereas the smallest proportion of athletes reported reductions exceeding 9% (2.86%). When stratified by sex, women more often reported a 5–7% reduction, while men more frequently reduced their body mass by 1–3%. These sex differences were not statistically significant (chi-square test of independence, *p* = 0.60). Regarding the timing of weight-cutting initiation, most respondents began the process 4–5 days before competition (31.43%), while the least common starting point was the day before the event (12.86%). Women more frequently than men initiated weight reduction 6–7 days prior to competition; however, this difference was also not statistically significant (chi-square test of independence, *p* = 0.58) (Table 3).

The most frequently used RWL methods before each competition included dehydration (71.01%, *n* = 49), restricting the intake of high-weight foods (68.57%, *n* = 48), and reducing the number of meals (65.71%, *n* = 46). The majority of athletes reported never using laxatives or diuretics (70.00%, *n* = 49), nor limiting dietary fiber intake (55.71%, *n* = 39). Additionally, 40.00% (*n* = 28) of respondents increased exercise intensity before every competition, while sauna use or hot baths were most commonly applied before approximately half of all competitions (25.71%, *n* = 18). Intensive training sessions performed with additional clothing were reported before every competition by 57.14% (*n* = 40) of the participants. A statistically significant difference between men and women was observed regarding the frequency of increasing exercise intensity (chi-square test of independence, *p* < 0.05). Detailed data on the use of specific RWL techniques among the study participants are presented in Table 4.

More than half of the respondents (81.16%, *n* = 56) restricted foods high in salt content during competition preparation, and a similarly high proportion limited products rich in simple sugars (75.71%, *n* = 53). The least commonly restricted food among judo athletes was meat (4.29%, *n* = 3). A statistically significant difference between men and women was observed regarding the restriction of fatty meat (chi-square test of independence, *p* < 0.001).

During the period of weight reduction to reach a lower weight category, the largest proportion of athletes reported increased consumption of lean meat (78.57%, *n* = 55) and low-calorie vegetables (71.43%, *n* = 50). Only a small fraction of respondents increased their intake of foods high in simple sugars (5.71%, *n* = 4). A significant difference between groups was found with respect to increasing the consumption of simple-sugar–rich products (*p* < 0.05). Most respondents indicated water as the most commonly consumed fluid during competition preparation (92.86%, *n* = 65), while fruit or fruit–vegetable smoothies were the least frequently chosen (7.14%, *n* = 5). Statistically significant sex differences were observed only in the consumption of isotonic drinks (chi-square test of independence, *p* < 0.001).

### 3.3. Physical and Psychological Effects of Rapid Weight Loss

The most frequently reported physical effects of RWL among respondents included reduced energy levels (42.86%, *n* = 30), general weakness (31.43%, *n* = 22), persistent hunger (28.57%, *n* = 20), and thirst (25.71%, *n* = 18). The highest proportion of athletes declared never experiencing heart palpitations (62.86%, *n* = 44), nausea during exercise (50.00%, *n* = 35), muscle tremors (50.00%, *n* = 35), or dizziness (42.86%, *n* = 30). No statistically significant differences were found between men and women in the frequency of experiencing physical consequences of rapid weight loss (chi-square test of independence, *p* > 0.05). Detailed information on the reported physical symptoms and their frequency is presented in Table 5.

The most commonly reported psychological consequences of RWL included difficulties with appetite control (37.14%, *n* = 26), low mood (28.57%, *n* = 20), and irritability (25.71%, *n* = 18). The largest proportion of athletes declared never experiencing an increase in motivation during periods of rapid weight reduction (41.43%, *n* = 29). No statistically significant differences were observed between men and women regarding the psychological effects of aggressive weight loss (chi-square test of independence, *p* > 0.05). Detailed data on the frequency of specific psychological symptoms are presented in Table 6.

More than half of the respondents reported consuming a meal composed of high-quality protein, complex carbohydrates, and fats between the weigh-in procedure and the fight (52.86%, *n* = 37). Fast food products were selected least frequently (11.43%, *n* = 8). No statistically significant differences were identified between men and women in the type of meal consumed during this period (chi-square test of independence, *p* = 0.69). Most athletes indicated isotonic drinks as their primary source of fluids between the weigh-in and the fight (74.29%, *n* = 52), whereas only a single respondent reported consuming beverages sweetened with artificial sweeteners (1.43%, *n* = 1). Likewise, differences between men and women in fluid choice did not reach statistical significance (chi-square test of independence, *p* = 0.05). A substantial majority of participants reported experiencing episodes of binge eating following the weigh-in (88.57%, *n* = 62). The frequency of this behavior did not differ significantly between men and women. Additionally, 70.00% (*n* = 49) of athletes noted discomfort during the fight related to post-weigh-in overeating; however, no significant sex differences were found (chi-square test of independence, *p* = 0.95). The most common symptoms associated with binge eating included a feeling of heaviness (60.00%, *n* = 42), decreased physical performance (48.57%, *n* = 34), and abdominal pain (34.29%, *n* = 24). A statistically significant sex difference was noted solely for digestive problems (*p* < 0.05). Discomfort related to insufficient energy intake during competition was reported by 67.14% of respondents (*n* = 47), with no significant differences between women and men (chi-square test of independence, *p* = 0.81). The most frequently reported consequences of under-eating included weakness (58.57%, *n* = 41), reduced muscle strength (52.86%, *n* = 37), and rapid fatigue (51.43%, *n* = 36). Headache was the only symptom for which a statistically significant difference between groups was observed (chi-square test of independence, *p* < 0.05).

### 3.4. Relationships Between Weight-Cutting Practices and Athletes’ Perceived Well-Being During Competition

Analysis of the associations between behaviors undertaken during rapid weight loss and the resulting physiological and psychological symptoms revealed several significant correlations. A moderate negative relationship was observed between athletes’ subjective evaluation of their body weight and the percentage of weight reduced before competition (Gamma = −0.33), suggesting that athletes who reduced a greater proportion of body mass tended to perceive their body weight more negatively. Moreover, a weak positive association was found between the percentage of body-weight reduction and the occurrence of binge eating after the weigh-in (Cramér’s V = 0.20), indicating that more aggressive weight loss may slightly increase the likelihood of compensatory overeating.

A strong positive relationship was identified between the use of dehydration techniques and the occurrence of dizziness (Gamma = 0.62), highlighting the substantial physiological burden associated with fluid restriction. Reducing the number of meals during weight cutting was associated with both concentration problems during the fight (Gamma = 0.42) and reduced motivation (Gamma = 0.54), suggesting that restrictive eating patterns may negatively affect cognitive performance and psychological readiness during competition. Dietary restrictions were additionally strongly linked to post-weigh-in binge eating episodes (Cramér’s V = 0.51), which may reflect a rebound response to prolonged food limitation.

Furthermore, a weak association was noted between the extent of body-weight reduction and discomfort during the fight related to insufficient energy intake (Cramér’s V = 0.17), indicating a modest but potentially meaningful impact of energy deficits on competition comfort. A very strong negative relationship was found between feelings of weakness during weight loss and the likelihood of binge eating after the weigh-in (Gamma = −0.75), suggesting that athletes experiencing greater weakness may be less able to engage in excessive food intake immediately after weigh-in. Finally, the type of meal consumed between the weigh-in and the fight showed a weak correlation with discomfort associated with overeating (Cramér’s V = 0.28), implying that meal composition may play a role in post-weigh-in gastrointestinal comfort. Detailed results are presented in Table 7.

## 4. Discussion

Interpretation of the present findings should consider methodological and contextual differences between judo and other combat sports frequently cited in the literature, such as mixed martial arts, wrestling, or taekwondo. These disciplines differ in competition regulations, frequency of weigh-ins, recovery time between weigh-in and competition, and sport culture, all of which may influence the choice and intensity of rapid weight reduction strategies. Due to the limited number of studies conducted exclusively in judo athletes, comparisons with other combat sports were necessary to provide a broader interpretative framework. Nevertheless, the high prevalence of dehydration, meal restriction, and post-weigh-in binge eating observed in the present study appears consistent with patterns reported in other weight-category sports, while the short recovery period typical of judo competitions may exacerbate the immediate physical and psychological consequences of these practices. Therefore, similarities and differences identified across combat sports should be interpreted cautiously, with emphasis on the specific competitive demands of judo.

Judo athletes, when reducing body mass to compete in a lower weight category, implement techniques that allow them to reach the target weight in the shortest possible time. Nevertheless, these interventions often affect the reduction in their athletic performance. Athletes most often do not take into account the potential negative effects of the techniques they use, and instead focus solely on effective reduction and achieving the desired body mass by the day of the weigh-in, which is associated not only with significant physical strain but also with psychological burden. This study allows for the identification of techniques that are most commonly used among judokas and for comparing them with the physical and psychological well-being of the athletes both during their application and during the fight. The results of the study may serve as a tool in selecting intensive weight reduction techniques in combat sports by increasing the awareness of athletes and coaches regarding their potential effects. This will allow for the planning of nutritional strategies that will minimize the risk of negative consequences of rapid weight loss that may affect fight performance.

The analysis showed that nearly all athletes use body mass reduction before competitions. The age at which such preparation began for most respondents ranged from 13 to 16 years. The participants, in order to qualify for a lower weight category, reduced on average 3.64 ± 1.74 kg, and for most respondents, this value constituted 5–7% of their initial body mass. Athletes most often began applying intensive reduction techniques 4–5 days before a competition.

The most commonly used RWL methods included dehydration, limiting the intake of high-volume foods, and reducing the number of meals. The products most commonly restricted during the reduction period were foods high in salt and those rich in simple sugars, whereas the products consumed in increased amounts were lean meat and low-calorie vegetables, and the dominant fluid during this period was water. Among the most frequently occurring physical complaints resulting from the applied RWL techniques were decreased energy, weakness, and chronic feelings of hunger and thirst. In terms of psychological aspects, athletes most often experienced difficulties with controlling appetite, mood decline, and irritability; moreover, the majority of athletes declared that episodes of binge eating occurred after the weigh-in procedure.

In the analysis conducted by Matthews and Nicholas involving MMA athletes, the average amount of reduced body mass was 5.6 ± 1.4 kg, which constituted 8.0 ± 1.8% of the initial body mass [14]. In contrast, in the study by Roklicer et al. performed among 229 wrestlers competing at the World Championships, the average number of reduced kilograms was 3.84 ± 2.82 to achieve the body mass required for competing in a lower weight category [12].

In the study by Drid et al., which included 199 participants from 20 countries who were elite sambo athletes, the mean level of reduction was 5.27 ± 7.57 kg. Among men, the average percentage of body mass loss was 8.49%, whereas in women it was 5.59%. Additionally, the average number of days before competition when athletes began the reduction was 11.87 ± 9.51 [15].

According to the recommendations of Januszko and Lange, total body mass loss should not exceed 5%. Based on these guidelines, reduction at this level may be implemented only if the time between the weigh-in procedure and the fight is sufficient (at least 3 h) for adequate refeeding and rehydration of the body [4]. Moreover, according to the position of the International Society of Sports Nutrition, in order to minimize health consequences and optimize athletic performance, a post-weigh-in body mass increase of 10% is recommended [16].

Discrepancies in the number of reduced kilograms may result from the characteristics of the sport disciplines of the study participants. The results of the present study are similar to those obtained by Roklicer et al. [12]. However, both in the present research and in the studies by Matthews and Nicholas [14], as well as in the analysis conducted by Drid et al. [15], the percentage of initial body mass exceeded recommended guidelines.

In an analysis by Seyhan involving 302 taekwondo athletes, it was found that competitors most often began reducing body mass 3–4 weeks before a competition or within the final 2 weeks preceding the event [17].

In the study by Artioli et al., which included 822 judokas, most athletes reported beginning RWL techniques ≤3 days before a competition; this response was declared by 37.2% of participants [18].

The findings of the present study are similar to those obtained by Artioli et al. [18], which may suggest that judo athletes initiate body mass reduction later than competitors in other weight-category sports. This may indicate that judokas overlook the aspect of gradual reduction involving decreases in fat mass and instead focus primarily on body mass losses resulting from water depletion, muscle glycogen reduction, and decreased gastrointestinal content.

In the study conducted by Ranisavljev et al., the mean age at which athletes began reducing body mass before competitions was 19.0 ± 5.2 years among men and 19.7 ± 2.7 years among women [19].

In the analysis by Reale et al., the mean age at which judokas initiated body mass reduction as part of competition preparation was 17.1 ± 5.5 years [20].

A study by Figlioli et al. carried out on 103 sambo athletes from 35 countries showed that the mean age of implementing body mass reduction during the preparatory period was 17.6 ± 4.7 years in senior athletes and 14.0 ± 3.9 among junior competitors [21].

In the study by Kons et al., which included 12 male judo athletes, the age at which athletes began practicing pre-competition weight reduction most often ranged between 13–16 years (*n* = 7) [22].

The age of initiation of RWL techniques in the present study is lower compared with the findings reported by the above-mentioned authors, but is consistent with the results of Kons et al. [22]. This discrepancy likely results from the characteristics of the sport, which may indicate that judo athletes begin reducing body mass at a younger age than athletes in other weight-category sports. Moreover, younger generations of combat sport athletes may begin their professional careers earlier, where pre-competition weight reduction constitutes a standard component of preparation.

In the study by Connor and Egan involving 30 male MMA athletes, fluid restriction was the most frequently used method of weight reduction. Among the participants, 62.1% (*n* = 29) declared that they always used this technique. Additionally, 72.4% (*n* = 29) never used laxatives, while 27.6% (*n* = 29) reported using a sauna before every competition. Nearly one-third of the athletes (34.5%; *n* = 29) stated that they always increased exercise intensity during rapid weight loss. Skipping one or two meals was practiced by 20.7% (*n* = 29) of the respondents [23].

The study by Castor-Praga et al. conducted on 160 combat sport athletes, including 112 taekwondo practitioners and 48 wrestlers, showed that the most frequently used RWL technique was increasing exercise intensity (33.59%), followed by training in plastic or thick clothing (30.35%) and fluid restriction (20.90%). Skipping meals before every competition was declared by 19.41% of the respondents, whereas the use of laxatives was reported by only 6.81% [24].

The analysis conducted by Janiszewska and Przybyłowicz, involving 192 taekwondo athletes competing at the Polish Taekwondo Championships, yielded similar results. The most frequently used RWL method was restricting the amount of food consumed (60.4%) and increasing the intensity of physical exercise (45.3%). Fluid restriction was indicated by 25.0% (*n* = 192) of the participants, whereas the use of laxatives was reported by only 2.1% (*n* = 192) [25].

In the study by Santos-Junior et al., which included 179 professional Brazilian MMA fighters, it was demonstrated that as RWL methods athletes predominantly used gradual dieting (64.2%, *n* = 115), increasing the number of training sessions (63.1%, *n* = 113), and restricting fluid intake (62.6%, *n* = 112) [26].

Another study on MMA athletes conducted by Ribas et al. indicated similar patterns. The most frequently used method was restricting fluid intake (72.0%, *n* = 18), using a sauna (60.0%, *n* = 15), gradual dieting (56.0%, *n* = 14), and increasing the number of training units (52.0%, *n* = 13) [27].

These results are consistent with the findings of the present study. Based on this, it can be stated that the frequency of using particular methods enabling rapid weight loss does not differ significantly among athletes of various combat sports. In the study by Nascimento et al., it was found that the greatest influence on the choice of weight-management methods among judokas came from coaches, both in beginner (88.2%) and advanced (76.9%) athletes, with parents and teammates also playing considerable roles [28]. This suggests that weight-cutting methods are deeply ingrained in combat sport environments and passed down to younger generations by coaches and more experienced athletes, which is why the findings of the present study align with those obtained by other researchers.

In the study conducted by Štangar et al., which included 138 male and female elite judo athletes, it was shown that the most frequently restricted products during pre-competition weight reduction were carbohydrate-rich foods (75.0%), salty foods (53.0%), and sugar-containing foods (52.0%). The products most commonly eliminated were sweetened beverages (58.0%) and foods high in fat (56.0%). In contrast, increased consumption of vegetables (51.0%) and protein-rich foods (45.0%) was observed [8].

The present study obtained similar results. The similarity of the findings may suggest that weight reduction in judo is based on repetitive patterns within this environment, which eliminate the same food products or macronutrients during the reduction period. Additionally, athletes may possess a comparable level of nutritional knowledge and draw information from similar sources. Relying on the same sources of information among athletes of different nationalities may result in similar strategies regarding the elimination of specific products, which consequently is reflected in comparable research outcomes obtained from athletes originating from various countries.

The fluid most frequently chosen by athletes in the period between the weigh-in and the fight was mineral water (76.0%) and sports drinks (70.0%). In this period, the meal most often consisted of high-carbohydrate products (89.0%) and high-protein foods (45.0%) [8].

When comparing the results of the present study with those obtained by Štangar et al., nutrition practices between the weigh-in and the fight did not differ substantially. In that study, the most frequently reported consequences of rapid weight loss were decreased energy levels (55.0%), increased aggressiveness and anger (34.0%), sleep disturbances (27.0%), lack of motivation or feelings of low mood (26.0%), as well as the desire to eat more despite limited possibilities (33.0%) [8].

In the study by Kurt et al. conducted on 159 Turkish judo athletes, the most common consequences of rapid weight loss included fatigue (56.0%, *n* = 89), increased thirst (52.8%, *n* = 84), and hunger (41.5%, *n* = 66). Furthermore, a high prevalence of reduced physical performance (41.5%, *n* = 66), reluctance to train (33.3%, *n* = 53), and lowered mood and irritability (31.4%, *n* = 50) was observed [29].

In the publication by Kouassi et al., based on individual interviews with 12 elite senior judo athletes from the Ivory Coast, competitors reported that dietary modifications introduced for the purpose of rapid weight loss drastically impair their performance and physical capabilities, and also lead to feelings of exhaustion. According to the authors, athletes are aware of the consequences of the practices they undertake [30]. From a psychological perspective, feelings associated with weight reduction differed markedly among participants. Some athletes reported high levels of stress and pressure related both to competition and achieving the target body weight, and after reaching it, they felt relief but also discouragement. Others stated that weight reduction was a motivating factor that strengthened their desire to win [30].

The publication by Carl et al. also emphasized that male athletes competing in sports with weight categories have a higher risk of developing eating disorders (18.0%) than endurance athletes (9.0%) or team sport athletes (5.0%). Moreover, they also show high rates of behaviors related to binge eating, restrictive dieting, or complete fasting [31].

Against the background of the findings of other researchers, participants in the present study experienced similar consequences of rapid weight loss. The consistency between the results of this study and those obtained by other authors likely arises from the use of similar nutritional strategies enabling rapid reductions in body mass, which may lead to the occurrence of comparable symptoms. Both the present study and research by other authors indicate, however, that the negative consequences of intensive weight-cutting techniques are far more prevalent than those exerting a positive influence on athletes.

### Strengths and Limitations

A strong aspect of this study is the specialized study group limited to a single discipline, which increases the practical applicability of the obtained data. The online format of the study made it possible to collect information from athletes training in clubs located in various regions of Poland, which minimized the influence of local conditions. Moreover, there is a limited number of studies that consider both the types of products restricted and those consumed in increased amounts, as well as the aspect of compensatory behaviors occurring after the weigh-in procedure.

The study also has certain limitations. There are several factors that may have influenced its results. The first of these is the level of nutritional knowledge of both the athletes and their coaches regarding pre-competition weight reduction, as well as the athletes’ experience in carrying it out. Another factor is the varied level of advancement among the athletes, which is associated with unequal access to professional dietary support. Furthermore, the study involved athletes of a single nationality, often training in the same clubs, and meeting during training camps or competitions. This facilitates the acquisition of information from similar sources and a comparable level of knowledge, which may contribute to the homogenization of body-mass–management strategies within this environment. The participants represented different sport levels and competed in events of varying prestige, which may influence the diversity of methods used. Additionally, the data obtained in the study are subject to errors related to subjective responses, which may be either overestimated or underestimated relative to reality. Moreover, the unequal sex distribution and overall sample size may limit the ability to detect small-to-moderate sex differences; therefore findings, particularly for between-sex comparisons, should be interpreted cautiously as the study is primarily powered to detect larger effects.

## 5. Conclusions

This study demonstrated that among judo athletes, body mass reduction prior to competition constitutes an integral part of pre-competition preparation and is a common practice. Athletes most frequently employ strategies such as dehydration, meal restriction, and increased training intensity. During weight-cutting preparations, the most commonly restricted food products are highly processed items rich in simple sugars, fats, and salt. At the same time, athletes increase their intake of low-calorie and high-protein products, such as vegetables and lean meat.

The findings suggest that the application of rapid weight-loss methods correlates with the occurrence of negative physical effects. Decreased energy levels, general weakness, constant hunger and thirst, as well as dizziness, were reported during the preparation period and during competitions. Weight reduction also has psychological consequences, causing mood deterioration, irritability, difficulties with concentration, and impaired appetite control. Additionally, substantial restriction in the number of consumed meals may negatively affect psychological well-being and athlete motivation.

A tendency toward excessive post-weigh-in overeating was also identified among the athletes. This phenomenon may be driven by a desire to compensate for the preceding period of severe dietary restriction, while simultaneously contributing to discomfort during the fight, manifesting as a feeling of heaviness, gastrointestinal complaints, and reduced physical performance. Insufficient energy intake, combined with binge-eating episodes after weigh-in, may impair performance during competition by limiting physical capacity and cognitive functions. These effects were found to occur regardless of gender.

The findings confirmed the research hypothesis that the nutritional strategies employed by athletes for rapid weight reduction have negative effects on both physical and psychological health and may impair performance during competition. Nevertheless, further research is necessary to better understand the mechanisms and factors determining the well-being of combat sport athletes during periods of intensive weight reduction, and to support the development of methods aimed at minimizing the risk of adverse outcomes.

## Figures and Tables

**Table 1:** BMI classification according to WHO [13].

BMI (kg/m^2^)	Interpretation of BMI
<18.5	Underweight
18.50–24.99	Body weight normal
25.00–29.99	Overweight
30.00–34.99	First-degree obesity
35.00–39.99	Second-degree obesity
≥40.00	Third-degree obesity

**Table 2:** Characteristics of the study group (*n* = 70).

Variable	Group	X	Med	Min	Max	SD	*p*-Value
Age [years]	Total (*n* = 70)	20.64	21.00	16.00	27.00	2.37	0.06
	Men (*n* = 53)	20.34	20.00	16.00	27.00	2.43	
	Women (*n* = 17)	21.59	21.00	19.00	25.00	1.94	
Height [cm]	Total (*n* = 70)	174.58	175.00	155.00	195.00	7.34	0.05
	Men (*n* = 53)	176.85	177.00	165.00	195.00	5.77	
	Women (*n* = 17)	167.50	170.00	155.00	179.00	7.33	
Body mass [kg]	Total (*n* = 70)	73.03	72.75	48.00	105.00	10.14	<0.001
	Men (*n* = 53)	75.43	75.50	50.00	105.00	9.52	
	Women (*n* = 17)	65.53	65.00	48.00	84.00	8.37	
BMI [kg/m^2^]	Total (*n* = 70)	23.88	23.96	18.37	31.35	2.36	0.22
	Men (*n* = 53)	24.08	24.21	18.37	31.35	2.52	
	Women (*n* = 17)	23.27	22.72	19.72	26.22	1.66	
Training experience [years]	Total (*n* = 70)	13.43	14.00	2.00	20.00	3.39	0.30
	Men (*n* = 53)	13.21	14.00	2.00	20.00	3.11	
	Women (*n* = 17)	14.12	15.00	5.00	20.00	4.17	

X—Mean, Med—Median, Min—Minimum, Max—Maximum, SD—Standard Deviation.

**Table 3:** Age at Initiation of Weight-Cutting, Weight-Loss Range, and Timing of Weight-Cutting Onset (*n* = 70).

	Group	<10 Years	10–12 Years	13–16 Years	16–18 Years	>18 Years	*p*-Value
Age at which athletes initiated weight-cutting	Total (*n* = 70)	3 (4.29%)	8 (11.43%)	46 (65.71%)	10 (14.28%)	3 (4.29%)	0.86
	Men (*n* = 53)	2 (3.77%)	6 (11.32%)	35 (66.04%)	8 (15.10%)	2 (3.77%)	
	Women (*n* = 17)	1 (5.88%)	2 (11.76%)	11 (64.71%)	2 (11.77%)	1 (5.88%)	
	**Group**	**1–3%**	**3–5%**	**5–7%**	**7–9%**	**>9%**	***p*** **-Value**
Weight-loss range	Total (*n* = 70)	15 (21.43%)	20 (28.57%)	22 (31.43%)	11 (15.71%)	2 (2.86%)	0.60
	Men (*n* = 53)	14 (26.42%)	14 (26.42%)	13 (24.53%)	10 (18.86%)	2 (3.77%)	
	Women (*n* = 17)	1 (5.88%)	6 (35.29%)	9 (52.94%)	1 (5.89%)	0 (0.00%)	
	**Group**	**1 Day Before Competition**	**2–3 Days Before**	**4–5 Days Before**	**6–7 Days Before**	**>7 Days Before**	***p*** **-Value**
Timing of weight-cutting onset	Total (*n* = 70)	9 (12.86%)	18 (25.71%)	22 (31.43%)	14 (20.00%)	7 (10.00%)	0.58
	Men (*n* = 53)	7 (13.21%)	14 (26.42%)	18 (33.96%)	8 (15.09%)	6 (11.32%)	
	Women (*n* = 17)	2 (11.76%)	4 (23.53%)	4 (23.53%)	6 (35.30%)	1 (5.88%)	

**Table 4:** Frequency of Rapid Weight-Loss (RWL) Techniques Used Before Competitions (*n* = 70).

Technique	Group	Never	Before 25% of Competitions	Before 50% of Competitions	Before 75% of Competitions	Before Every Competition	*p*-Value
Dehydration (fluid restriction)	Total (*n* = 70)	4 (5.80%)	7 (10.14%)	2 (2.90%)	7 (10.15%)	49 (71.01%)	0.87
	Men (*n* = 53)	4 (7.69%)	5 (9.62%)	2 (3.85%)	4 (7.69%)	37 (71.15%)	
	Women (*n* = 17)	0 (0.00%)	2 (11.76%)	0 (0.00%)	3 (17.65%)	12 (70.59%)	
Reduction in the number of meals	Total (*n* = 70)	3 (4.29%)	1 (1.43%)	9 (12.86%)	11 (15.71%)	46 (65.71%)	0.49
	Men (*n* = 53)	2 (3.77%)	1 (1.89%)	8 (15.09%)	5 (9.44%)	37 (69.81%)	
	Women (*n* = 17)	1 (5.88%)	0 (0.00%)	1 (5.88%)	6 (35.29%)	9 (52.95%)	
Avoiding salt	Total (*n* = 70)	17 (24.29%)	7 (10.00%)	5 (7.14%)	13 (18.57%)	28 (40.00%)	1.00
	Men (*n* = 53)	13 (24.53%)	4 (7.55%)	5 (9.43%)	10 (18.87%)	21 (39.62%)	
	Women (*n* = 17)	4 (23.53%)	3 (17.65%)	0 (0.00%)	3 (17.65%)	7 (41.17%)	
Avoiding high-weight foods	Total (*n* = 70)	6 (8.57%)	2 (2.86%)	10 (14.29%)	4 (5.71%)	48 (68.57%)	0.76
	Men (*n* = 53)	6 (11.32%)	1 (1.89%)	7 (13.21%)	3 (5.66%)	36 (67.92%)	
	Women (*n* = 17)	0 (0.00%)	1 (5.88%)	3 (17.65%)	1 (5.88%)	12 (70.59%)	
Limiting fiber intake	Total (*n* = 70)	39 (55.71%)	3 (4.29%)	6 (8.57%)	6 (8.57%)	16 (22.86%)	0.22
	Men (*n* = 53)	27 (50.94%)	3 (5.66%)	5 (9.43%)	4 (7.55%)	14 (26.42%)	
	Women (*n* = 17)	12 (70.59%)	0 (0.00%)	1 (5.88%)	2 (11.77%)	2 (11.76%)	
Increased intake of low-weight foods	Total (*n* = 70)	9 (12.86%)	6 (8.57%)	6 (8.57%)	14 (20.00%)	35 (50.00%)	0.39
	Men (*n* = 53)	9 (16.98%)	4 (7.55%)	4 (7.55%)	10 (18.87%)	26 (49.06%)	
	Women (*n* = 17)	0 (0.00%)	2 (11.76%)	2 (11.76%)	4 (23.53%)	9 (52.94%)	
Increased training intensity	Total (*n* = 70)	11 (15.71%)	6 (8.57%)	13 (18.57%)	12 (17.15%)	28 (40.00%)	<0.05 *
	Men (*n* = 53)	7 (13.21%)	3 (5.66%)	8 (15.09%)	9 (16.98%)	26 (49.06%)	
	Women (*n* = 17)	4 (23.53%)	3 (17.65%)	5 (29.41%)	3 (17.65%)	2 (11.76%)	
Use of sauna or hot baths	Total (*n* = 70)	13 (18.57%)	17 (24.29%)	18 (25.71%)	7 (10.00%)	15 (21.43%)	0.13
	Men (*n* = 53)	11 (20.75%)	15 (28.30%)	13 (24.53%)	3 (5.66%)	11 (20.75%)	
	Women (*n* = 17)	2 (11.76%)	2 (11.76%)	5 (29.41%)	4 (23.53%)	4 (23.53%)	
Intensive exercise with additional clothing	Total (*n* = 70)	2 (2.86%)	5 (7.14%)	10 (14.29%)	13 (18.57%)	40 (57.14%)	0.15
	Men (*n* = 53)	2 (3.77%)	3 (5.66%)	5 (9.43%)	10 (18.87%)	33 (62.26%)	
	Women (*n* = 17)	0 (0.00%)	2 (11.76%)	5 (29.41%)	3 (17.65%)	7 (41.18%)	
Use of laxatives or diuretics	Total (*n* = 70)	49 (70.00%)	9 (12.86%)	4 (5.71%)	8 (11.43%)	0 (0.00%)	0.40
	Men (*n* = 53)	39 (73.58%)	6 (11.32%)	2 (3.77%)	6 (11.33%)	0 (0.00%)	
	Women (*n* = 17)	10 (58.82%)	3 (17.65%)	2 (11.76%)	2 (11.77%)	0 (0.00%)	

* = *p* < 0.05

**Table 5:** Physical effects of rapid weight loss (*n* = 70).

Physical Effect	Group	Never	Before 25% of Competitions	Before 50% of Competitions	Before 75% of Competitions	Always	*p*-Value
Reduced energy	Total (*n* = 70)	2 (2.86%)	7 (10.00%)	15 (21.43%)	16 (22.86%)	30 (42.85%)	0.36
	Men (*n* = 53)	1 (1.89%)	7 (13.21%)	10 (18.87%)	9 (16.97%)	26 (49.06%)	
	Women (*n* = 17)	1 (5.88%)	0 (0.00%)	5 (29.41%)	7 (41.18%)	4 (23.53%)	
Weakness	Total (*n* = 70)	3 (4.29%)	12 (17.14%)	13 (18.57%)	20 (28.57%)	22 (31.43%)	0.83
	Men (*n* = 53)	2 (3.77%)	9 (16.98%)	12 (22.64%)	13 (24.53%)	17 (32.08%)	
	Women (*n* = 17)	1 (5.88%)	3 (17.65%)	1 (5.88%)	7 (41.18%)	5 (29.41%)	
Sleep problems	Total (*n* = 70)	24 (34.29%)	18 (25.71%)	10 (14.29%)	18 (25.71%)	0 (0.00%)	0.12
	Men (*n* = 53)	21 (39.62%)	12 (22.64%)	9 (16.98%)	11 (20.76%)	0 (0.00%)	
	Women (*n* = 17)	3 (17.65%)	6 (35.29%)	1 (5.88%)	7 (41.18%)	0 (0.00%)	
Headaches	Total (*n* = 70)	31 (44.29%)	17 (24.29%)	11 (15.71%)	6 (8.57%)	5 (7.14%)	0.34
	Men (*n* = 53)	25 (47.17%)	14 (26.42%)	6 (11.32%)	3 (5.66%)	5 (9.43%)	
	Women (*n* = 17)	6 (35.29%)	3 (17.65%)	5 (29.41%)	3 (17.65%)	0 (0.00%)	
Dizziness	Total (*n* = 70)	30 (42.86%)	22 (31.43%)	12 (17.14%)	4 (5.71%)	2 (2.86%)	0.68
	Men (*n* = 53)	24 (45.28%)	16 (30.19%)	7 (13.21%)	4 (7.55%)	2 (3.77%)	
	Women (*n* = 17)	6 (35.29%)	6 (35.29%)	5 (29.41%)	0 (0.00%)	0 (0.00%)	
Muscle tremors	Total (*n* = 70)	35 (50.00%)	16 (22.86%)	11 (15.71%)	6 (8.57%)	2 (2.86%)	0.47
	Men (*n* = 53)	26 (49.06%)	11 (20.75%)	8 (15.09%)	6 (11.32%)	2 (3.78%)	
	Women (*n* = 17)	9 (52.94%)	5 (29.42%)	3 (17.65%)	0 (0.00%)	0 (0.00%)	
Nausea during exercise	Total (*n* = 70)	35 (50.00%)	20 (28.57%)	6 (8.57%)	3 (4.29%)	6 (8.57%)	0.05
	Men (*n* = 53)	23 (43.40%)	16 (30.19%)	5 (9.43%)	3 (5.66%)	6 (11.32%)	
	Women (*n* = 17)	12 (70.59%)	4 (23.53%)	1 (5.88%)	0 (0.00%)	0 (0.00%)	
Heart palpitations	Total (*n* = 70)	44 (62.86%)	15 (21.43%)	7 (10.00%)	0 (0.00%)	4 (5.71%)	0.84
	Men (*n* = 53)	35 (66.04%)	7 (13.21%)	7 (13.21%)	0 (0.00%)	4 (7.54%)	
	Women (*n* = 17)	9 (52.94%)	8 (47.06%)	0 (0.00%)	0 (0.00%)	0 (0.00%)	
Constant hunger	Total (*n* = 70)	7 (10.00%)	16 (22.86%)	17 (24.29%)	10 (14.29%)	20 (28.56%)	0.57
	Men (*n* = 53)	7 (13.21%)	12 (22.64%)	8 (15.09%)	8 (15.09%)	18 (33.97%)	
	Women (*n* = 17)	0 (0.00%)	4 (23.53%)	9 (52.94%)	2 (11.77%)	2 (11.76%)	
Constant thirst	Total (*n* = 70)	6 (8.57%)	18 (25.71%)	14 (20.00%)	14 (20.00%)	18 (25.72%)	0.71
	Men (*n* = 53)	6 (11.32%)	14 (26.42%)	9 (16.98%)	9 (16.98%)	15 (28.30%)	
	Women (*n* = 17)	0 (0.00%)	4 (23.53%)	5 (29.41%)	5 (29.41%)	3 (17.65%)	

**Table 6:** Psychological effects of rapid weight loss (*n* = 70).

Psychological Effect	Group	Never	Before 25% of Competitions	Before 50% of Competitions	Before 75% of Competitions	Always	*p*-Value
Irritability	Total (*n* = 70)	6 (8.57%)	7 (10.00%)	11 (15.71%)	28 (40.00%)	18 (25.71%)	0.46
	Men (*n* = 53)	5 (9.43%)	5 (9.43%)	10 (18.87%)	20 (37.74%)	13 (24.53%)	
	Women (*n* = 17)	1 (5.88%)	2 (11.76%)	1 (5.88%)	8 (47.06%)	5 (29.41%)	
Concentration problems	Total (*n* = 70)	12 (17.14%)	9 (12.86%)	22 (31.43%)	15 (21.43%)	12 (17.14%)	0.14
	Men (*n* = 53)	10 (18.87%)	9 (16.98%)	17 (32.08%)	7 (13.21%)	10 (18.87%)	
	Women (*n* = 17)	2 (11.76%)	0 (0.00%)	5 (29.41%)	8 (47.06%)	2 (11.76%)	
Low mood/depressive feelings	Total (*n* = 70)	11 (15.71%)	12 (17.14%)	11 (15.71%)	16 (22.86%)	20 (28.57%)	0.65
	Men (*n* = 53)	9 (16.98%)	9 (16.98%)	9 (16.98%)	11 (20.75%)	15 (28.30%)	
	Women (*n* = 17)	2 (11.76%)	3 (17.65%)	2 (11.76%)	5 (29.41%)	5 (29.41%)	
Lack of motivation	Total (*n* = 70)	20 (28.57%)	20 (28.57%)	12 (17.14%)	12 (17.14%)	6 (8.57%)	0.74
	Men (*n* = 53)	15 (28.30%)	15 (28.30%)	9 (16.98%)	8 (15.09%)	6 (11.32%)	
	Women (*n* = 17)	5 (29.41%)	5 (29.41%)	3 (17.65%)	4 (23.53%)	0 (0.00%)	
Increase in motivation	Total (*n* = 70)	29 (41.43%)	13 (18.57%)	17 (24.29%)	6 (8.57%)	5 (7.14%)	0.23
	Men (*n* = 53)	25 (47.17%)	9 (16.98%)	10 (18.87%)	5 (9.43%)	4 (7.55%)	
	Women (*n* = 17)	4 (23.53%)	4 (23.53%)	7 (41.18%)	1 (5.88%)	1 (5.88%)	
Appetite dysregulation/increased urge to eat	Total (*n* = 70)	15 (21.43%)	16 (22.86%)	4 (5.71%)	9 (12.86%)	26 (37.14%)	0.96
	Men (*n* = 53)	13 (24.53%)	9 (16.98%)	3 (5.66%)	8 (15.09%)	20 (37.74%)	
	Women (*n* = 17)	2 (11.76%)	7 (41.18%)	1 (5.88%)	1 (5.88%)	6 (35.29%)	

**Table 7:** Associations between weight-cutting behaviors and physiological and psychological symptoms among athletes.

Variable 1	Variable 2	Coefficient Type	Value	Strength and Direction of Association
Subjective assessment of body weight	Percentage of body-weight reduction	Gamma	−0.33	moderate, negative
Percentage of body-weight reduction	Binge eating after weigh-in	Cramér’s V	0.20	weak, positive
Use of dehydration	Dizziness	Gamma	0.62	strong, positive
Reduction in number of meals	Concentration problems	Gamma	0.42	moderate, positive
Reduction in number of meals	Lack of motivation	Gamma	0.54	strong, positive
Reduction in number of meals	Binge eating after weigh-in	Cramér’s V	0.51	strong, positive
Percentage of body-weight reduction	Fight-related discomfort due to under-eating	Cramér’s V	0.17	weak, positive
Weakness during weight-cutting	Binge eating after weigh-in	Gamma	−0.75	very strong, negative
Type of meal consumed between weigh-in and fight	Discomfort related to overeating	Cramér’s V	0.28	weak, positive

## Data Availability

The original contributions presented in this study are included in the article. Further inquiries can be directed to the corresponding author.
